# Citrus alkaline extracts improve LPS-induced pulmonary fibrosis via epithelial mesenchymal transition signals

**DOI:** 10.1186/s13020-023-00766-0

**Published:** 2023-05-29

**Authors:** Li Junjie, Gu Cheng, Luo Kangkang, Li Yu, Yuan Zhiyao, Wu Xudong, Zhou Xianmei, Lu Xiaomin

**Affiliations:** 1 State Key Laboratory of Pharmaceutical Biotechnology, School of Life Sciences, Nanjing University, 163 Xianlin Avenue, Nanjing, 210023 China; 2 Jiangsu Province Hospital of Chinese Medicine, Affiliated Hospital of Nanjing University of Chinese Medicine, 155 Hanzhong Road, Nanjing, 210004 China; 3 Nanjing Stomatological Hospital, Medical School of Nanjing University, Nanjing, 210008 China

**Keywords:** Citrus alkaline extracts (CAE), Pulmonary fibrosis, Epithelial-mesenchymal transition (EMT), Wnt/β-catenin, STATs

## Abstract

**Background:**

Acute respiratory distress syndrome (ARDS) is a serious life threatening clinical critical illness. ARDS-related pulmonary fibrosis is a common complication of ARDS. The occurrence of early pulmonary fibrosis indicates a higher incidence and mortality of multiple organ failure. LPS-induced ARDS-related pulmonary fibrosis model in mice was established in this study. And we have explored the anti-pulmonary fibrosis effects and molecular mechanisms of the Citrus Alkaline Extracts (CAE) in vivo and in vitro.

**Methods:**

Pulmonary fibrosis mouse model and lung epithelial cell injury model were established in this study. H&E, Masson and Sirius Red staining were used to estimate lung tissue damage. Immunohistochemistry and western blotting were used to analyze proteins expression. Protein-protein interaction was observed by Co-Immunoprecipitation. Systemic impact of CAE on signaling pathway was examined by RNA-seq.

**Results:**

Through H&E, Masson and Sirius Red staining, it was convincingly indicated that therapeutic administration of CAE alleviated lung injury and fibrosis, while pretreated administration of CAE showed weak improvement. In vitro experiments showed that CAE had dual regulation to E-cadherin and N-cadherin, the important indicators of epithelial-mesenchymal transition (EMT). And it was further demonstrated that CAE reversed TGF-β1-induced EMT mainly through Wnt/β-catenin, Stat3/6 and COX2/PGE2 signals. Through RNA-Seq, we discovered important mechanisms by which CAE exerts its therapeutic effect. And network pharmacology analysis demonstrated core potential targets of CAE in EMT.

**Conclusion:**

Thus, this study provides new therapeutic effects of CAE in anti-fibrosis, and offers potential mechanisms for CAE in LPS-induced pulmonary fibrosis.

**Graphical Abstract:**

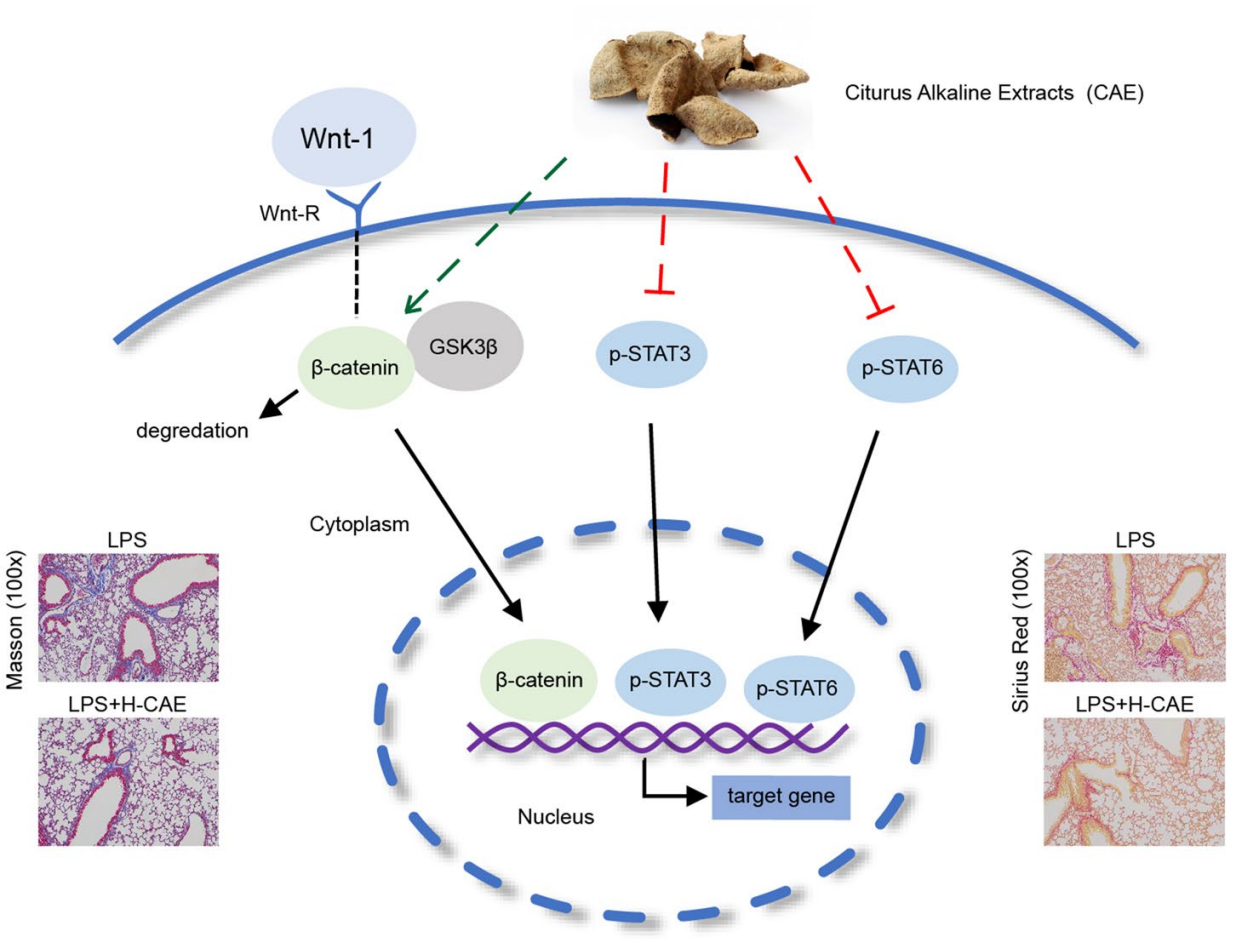

**Supplementary Information:**

The online version contains supplementary material available at 10.1186/s13020-023-00766-0.

## Introduction

Acute respiratory distress syndrome (ARDS) is the acute refractory hypoxic respiratory insufficiency characterized by diffuse edema of pulmonary interstitial and alveolar, which is caused by the damage of lung capillary endothelial cells and alveolar epithelial cells in the process of non-cardiogenic diseases such as severe infection, shock, trauma, and burns. No matter SARS virus, avian influenza virus, or bacterial infection, it may cause severe pneumonia that seriously endangers human health. Among them, infection is the most common cause of ARDS, and its secondary pulmonary fibrosis has become an important cause of poor prognosis [1]. Even the surviving patients will have a negative impact on the quality of life because of pulmonary fibrosis. Therefore, exploring the mechanism of LPS-induced ARDS-related pulmonary fibrosis and possible intervention treatments has important clinical significance.

The damage and repair of epithelial cells and the transformation between cells play a key role in the occurrence and development of pulmonary fibrosis. Many studies have shown that epithelial cells acquire a mesenchymal cell phenotype through EMT and it became an important source of fibroblasts and myofibroblasts. EMT was considered to be a new model for the occurrence and development of pulmonary fibrosis [2]. In this process, TGF-β1 is the most important inducer. It activates a series of transcription factors, thereby regulating the transcription of downstream EMT-related genes and inducing the transformation of epithelial cells to mesenchymal cells [3]. Alveolar epithelial cells that are stimulated by injury appear morphological changes such as changes from cubic cells to elongated or spindle-shaped cells, loss of epithelial characteristic markers such as E-cadherin [4]. When lung tissue is injured, the coordination of epithelial cells, mesenchymal cells, and extracellular matrix is a necessary process for the self-repair response of lung tissue after damage. When this repair is out of control, the epithelial tissue structure will disappear, myofibroblasts will be activated, and fibroblasts will accumulate, which will lead to the occurrence of pulmonary fibrosis [5]. Therefore, inhibiting the EMT process and regulating the direction of differentiation of alveolar epithelial cells has important value in the prevention and treatment of LPS-induced ARDS-related pulmonary fibrosis.

Current mechanism analysis demonstrate that Wnt can crosstalk with other pro-fibrotic growth factors such as TGF-β, and the increase level of β-catenin in nucleus during the fibroproliferative phase after acute lung injury is also identified [6, 7]. Moreover, the aberrant activation of Wnt/β-catenin pathway ultimately triggers distinct epithelial regeneration at the bronchiolar-alveolar junction and EMT, resulting in severe and irreversible lung tissue remodeling [8]. Besides, STATs and Cyclooxygenase 2 (COX2) / Prostaglandin E2 (PEG2) signals also play a key role in the development of pulmonary fibrosis [9, 10].

The treatment of pulmonary fibrosis, especially ARDS-related pulmonary fibrosis, currently lacks effective drugs. In the past ten years, hormone therapy has been used to treat severe viral pneumonia, but pulmonary fibrosis is still a fatal complication, and hormones cannot improve the prognosis [11]. Therefore, based on the mechanism of the occurrence and development of pulmonary fibrosis, finding effective drugs with low side effects is still a problem to be solved in the treatment of ARDS. To this end, we proceed from traditional Chinese medicine to carry out long-term explorations. Citrus belongs to the rutaceae and it has been proved that rutaceae has anti-fibrostic, anti-inflammatory effects [12, 13]. In this study, we examined the effect of Citrus Alkaline Extracts (CAE) on LPS-induced pulmonary fibrosis in mice and explored the possible mechanism involved. The composition of CAE which mainly contains flavonoids, volatile oils, and alkaloids has been identified and reported, which is also an important basis for this research [14].

## Materials and methods

### Preparation of CAE

The dried *Citrus* (1000 g) was extracted three times with 75% ethanol for 3 h (3000 mL × 3) under reflux. The filtrate was concentrated under decompression. The collected mass was dissolved in distilled water (300 mL), then adjusted to a pH of 2 with HCl (20%, g/g) and washed with ethyl acetate (300 mL × 3) to remove acidic compositions. The rest water part was adjusted pH to 9 using ammonia water (23%, g/g), and separated with ethyl acetate (300 mL × 3). The combined ethyl acetate was dried with anhydrous sodium sulfate and evaporated under decompression to generate CAE (1.8% yield). Some components of CAE—N-Methyltyramine, Synephrine, Flavanone, Hesperitin, Limonin, Narirutin, Hesperidin, Tangeretin and Sinensetin—and other ingredients were identified by LC-MS (4600 UPLC/Triple TOF) and shown in Additional file 1.

### A murine model of LPS-induced pulmonary fibrosis

Male C57BL/6 mice are kept at room temperature (25 ± 1 °C), atmospheric humidity (50% ± 10%), under a regular 12-hour dark light cycle, and fed with standard laboratory food and water. Animal welfare and experimental procedures were carried out strictly in accordance with the Guide for the Care and Use of Laboratory Animals (National Institutes of Health, the United States) and the related ethical regulations of our university. Animal studies were in compliance with the ARRIVE guidelines [15].

**Fig. 1:**
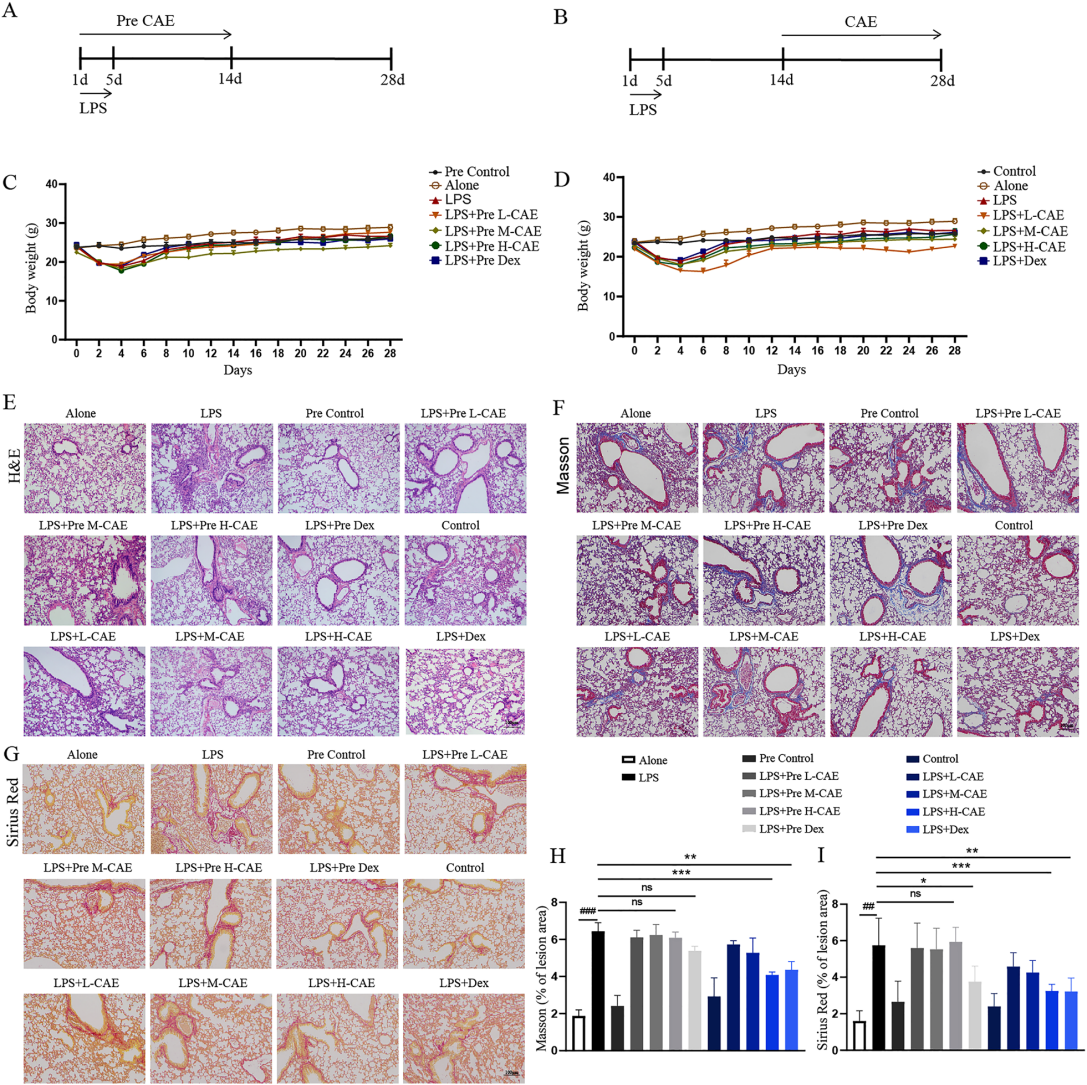
CAE ameliorated LPS- induced lung injury and pulmonary fibrosis. **A**, **B** Time chart of mouse model building. **A** Administration procedure of the pretreated groups. **B** Administration procedure of the therapeutic groups. **C**,** D** Mice body weight change. **E** H&E staining. **F** Masson staining. **G **Sirius Red staining. The scale bar is 100 μm. **H**, **I** Area density analysis of Masson’s trichrome and Sirius Red staining, respectively. Results were shown as the means ± SEM (n = 8). *P< 0.05, **P < 0.01, ***P < 0.001 vs. the LPS group, ^#^ P < 0.05, ^##^ P < 0.01, ^###^ P < 0.001 vs. Alone group

The pulmonary fibrosis model was established by intraperitoneal injection of purified LPS extracted from the membrane of *Escherichia coli* 0111:B4 (Sigma-Aldrich, St. Louis, MO, USA) at 5 mg kg^− 1^ day^− 1^ in a total volume of 50 µl from the 1st to the 5th day [11]. The preliminary experiment revealed that 5-day-injected LPS caused pulmonary fibrosis on the 14th day (data not shown), and then we chose the 14th day as a separation. CAE administration was categorized into pretreated groups (gavaged from the 1st to the 14th day, and shown as Pre) and therapeutic groups (gavaged from the 15th to the 28th day). Dexamethasone (Dex) was used as positive control. Details are as follow: Ninety-six of 8-week-old C57BL/6 male mice weighing 18–22 g were randomly divided into 12 groups: Alone (normal saline), LPS (5 mg kg^−1^ day^−1^), Pre Control (0.5% CMC-Na for the former 14 days), LPS + Pre H-CAE (96 mg kg^−1^ day^−1^, pretreated), LPS + Pre M-CAE (64 mg kg^−1^ day^−1^, pretreated), LPS + Pre L-CAE group (32 mg kg^−1^ day^−1^, pretreated), LPS + Pre Dex (5 mg kg^−1^ day^−1^, pretreated), Control (0.5% CMC-Na for the later 14 days), LPS + H-CAE (96 mg kg^−1^ day^−1^), LPS + M-CAE (64 mg kg^−1^ day^−1^), LPS + L-CAE group (32 mg kg^−1^ day^−1^), LPS + Dex (5 mg kg^−1^ day^−1^). And after 28 days, all of the mice were sacrificed. The method of CAE administration was shown in Fig. 1A and B. Part of the lung tissue was fixed with 10% formalin to prepare paraffin, and other parts were quick-frozen with liquid nitrogen and stored at −80 °C for later use. Dex was purchased from Sigma-Aldrich (St. Louis, MO, USA). And CAE was extracted, separated and identified in laboratory as described previously [16].

### Tissue sectioning and histopathology

Lung specimens fixed in 10% buffered formalin and were embedded in paraffin blocks. Paraffin sections of tissues were placed on glass slides, and paraffin was removed with xylene. The slices were handed over to Service B io Technology Co., Ltd (Wuhan, China) for serial staining include Hematoxylin and Eosin, Masson’s Trichrome and Sirius Red staining to evaluate the severity of fibrosis. Examinations were performed and photographs were captured with a light microscope. ImageJ software (National Institutes of Health, Maryland, USA) was used to calculate the ratio of the area with positive expression to the total field of Masson’s Trichrome and Sirius Red staining.

### Immunohistochemistry staining

Lung specimens fixed in 10% buffered formalin and were embedded in paraffin blocks. Paraffin-embedded lung sections were heat-fixed, deparaffinized, rehydrated, antigen retrieval, blocked with 3% goat serum and incubated with anti-α-SMA antibody (1:200, Cell Signaling Technology, MA) or anti-COL1A1 antibody (1:200, Cell Signaling Technology, MA) or anti-E-cadherin antibody (1:150, Cell Signaling Technology, MA) or anti-N-cadherin antibody (1:150, Cell Signaling Technology, MA) overnight at 4 °C, then the slides were detected using Real Envision Detection kit (GeneTech, Shanghai, China) according to the manufacturer’s instructions. Observe the sections with a microscope and take pictures. Image J software calculated the ratio of positive expression area to the total field of immunohistochemical staining of α-SMA, COL1A1, E-cadherin and N-cadherin.

### Measurement of collagen I and hydroxyproline contents

The contents of collagen I and hydroxyproline in lung tissues were determined by commercially available kits obtained from Nanjing Jiancheng Bioengineering Inc. (Catalog #H589, Catalog #A030-2-1). Hydrolysate (1 mL) was added to serum (500 µL) or lung tissue (50 mg, wet weight) and incubated in water at 95 °C for 20 min for hydrolysis. The sample solution was adjusted to a pH of 6.0 to 6.8 using the pH modulation A and B solution in the kit. Double distilled water was added to 10 mL and took 3 mL to add 20 mg of carbon, then centrifuged and retained the supernatant. The detection solution sequentially added in the enzyme plate according to the operation table of the instructions and incubated in water at 60 °C for 15 min and centrifuged after cooling. The supernatant was determined the absorbance at a wavelength of 550 nm.

### Cell culture

A549 (human lung adenocarcinoma) cell line was obtained from the China Center for Type Culture Collection (Wuhan, China) and was cultured in 25-cm^2^ flasks (Corning, Corning, NY, USA) containing Dulbecco’s modified Eagle medium (DMEM; Gibco, Thermo Fisher Scientific, Waltham, MA), containing 10% fetal bovine serum (FBS; Gibco, Thermo Fisher Scientific, Waltham, MA) and 1% penicillin streptomycin (Biological Industries, Haemek, IsCAEl), at 5% CO_2_ and 37 °C. To create the in vitro fibrosis stress model, A549 cells were treated with TGF-β1 (10 ng·mL^−1^, Pepro Tech, USA) for 48 h while which they were treated with three different doses of CAE (250 µg mL^−1^, 500 µg mL^−1^ and 1000 µg mL^−1^) or sivelestat sodium (100 µg mL^−1^, Catalog #C105530, Chemegen, USA) [17, 18] .

### CCK-8 assay

Cells were seeded in 96-well plate and incubated with various doses of CAE for 48 h. And then added 20 µL CCK-8 Antibody Blocking Peptide (Bioss, Beijing, China) to each well, placed the culture plate in the incubator for 4 h. Finally, the absorbance at 450 nm were measured with a microplate reader. The survival rate of cells was calculated based on the absorbance value of each well.

### Western blot analysis

A549 cells were seeded in a 6-well plate, the treatment groups were given different doses of CAE (250 µg mL^−1^, 500 µg mL^−1^ and 1000 µg mL^−1^) and TGF-β1 (10 ng mL^−1^), while the model group was given TGF-β1 (10 ng mL^−1^) and the control group was given the same volume of DMSO as the CAE after cell adherence. After stimulating and culturing for 48 h, cells were collected and lysed using RIPA Lysis Buffer (Beyotime, Shanghai, China) and quantitated by BCA Protein Assay Kit (Beyotime, Shanghai, China), obtained protein lysates were degenerated at 100 ℃ for 5 min and separated by 10% SDS-PAGE and electrophoretically transferred onto poiyvinylidene fluoride (PVDF) membranes (Millipore Corp, Bedford, MA). The membranes were blocked with 3% BSA (BioFroxx, Guangzhou, China) for 1 h at room temperature, then incubated with specific primary antibodies including anti-E-cadherin antibody (1:1000, Cell Signaling Technology, MA) or anti-N-cadherin antibody (1:1000, Cell Signaling Technology, MA) or anti-α-SMA antibody (1:1000, Cell Signaling Technology, MA) or anti-p-stat3 antibody (1:1000, Cell Signaling Technology, MA) or anti-p-stat6 antibody (1:1000, Cell Signaling Technology, MA) or anti-β-catenin antibody (1:1000, Cell Signaling Technology, MA) or anti-Wnt-1 antibody (1:1000, Santa Cruz Biotechnology) or anti-COX2 antibody (1:1000, Abcam, CA) overnight at 4 ℃, and then incubated with a horseradish peroxidase (HRP) coupled secondary antibody. Protein bands were visualized using Western blotting detection system according to the manufacturer’s instructions. Image J software calculated the gray-scale value of the bands.

### CHIP assay

Crosslinking DNA and proteins in A549 cells using 1% formaldehyde solution under physiological conditions. Chromatin was broken down by ultrasound, followed by the addition of anti-EZH2 antibody for positive control, anti-IgG antibody for negative control, or anti-Egr1 antibody precipitated crosslinked complex. DNA fragments bound to antibodies were precipitated. Perform de-crosslinking to purify DNA fragments. DNA sequences specifically bound to antibodies are screened by RT-qPCR. The reverse (5′GGAAATGGCTCTGGACTTGGCGGTA3′) and forward (5′GGAGGCAGCCGTTCGGAGGATTATT3′) pair of primers was used to amplify PTEN promoter. The reverse (5′TTGGTGGTTGGGTGATGGAG3′) and forward (5′GATTGCAAGCCCCAATCCC3′) pair of primers was used to amplify COL1A1 promoter.

### Immunofluorescence staining

A549 cells were cultured at 24-well plate and TGF-β1 (10 ng mL^−1^) was added in a medium of cells with CAE (500 µg mL^−1^, 1000 µg mL^−1^) for 48 h. Then the medium was aspirated and the cells were gently shaken with PBS buffer. The cells were fixed in 1% paraformaldehyde for 30 min and were permeabilized with 0.1% Triton X-100 in PBS for 20 min at room temperature. Nonspecific binding sites were blocked by incubating the cells with 3% goat serum for 1 h. The fixed cells were incubated with a primary antibody against N-cadherin (1:200, Cell Signaling Technology, MA) or E-cadherin (1:200, Cell Signaling Technology, MA) overnight at 4 °C, followed by incubation with fluorescence (FITC)-conjugated goat anti-rabbit IgG (1:1000, Thermo Fisher Scientific, USA) for 1 h at room temperature. The cellular nuclei were stained with DAPI for 15 min. All samples were imaged with a light microscope. Image J software (NIH) was used to calculate the ratio of the area with positive expression to the total field of N-cadherin and E-cadherin.

### Immunoprecipitation studies

A549 cells were cultured at 10 cm dish and treated with 1000 µg mL^−1^ CAE in the presence of 10 ng mL^−1^ TGF-β1, while the model group was given TGF-β1 and the control group was added same volume of DMSO as the CAE. Then the culture dishes were placed in an incubator for 48 h (with 5% CO_2_ and 37 °C). A small part of the harvested cell lysates was used for western blot, and the rest of the lysates was incubated with 4 µg GSK3β antibody (1:1000, Proteintech, USA) at 4 °C overnight and precipitated with Protein A/G Magnetic Beads (Thermo Fisher Scientific, USA) for another 1 h at room temperature. The beads were washed 4 times with washing buffer (PBS buffer with 0.05‱ Tween 20) on the magnetic stand and the immunoprecipitated proteins were boiled for 10 min in loading buffer. Finally, the immunoprecipitated proteins were detected by western blot.

### SOD, MDA and NO assay

A549 Cells seeded in 6-well plate and the supernatant of cells were collected respectively after 48 h. Cells were sonicated by ultrasonic disruptor and quantitated by BCA Protein Assay Kit. Superoxide Dismutase (SOD) assay kit (WST-1 method; Nanjing Jiancheng Bioengineering Inc.), Cell Malondialdehyde (MDA) assay kit (Colorimetric method; Nanjing Jiancheng Bioengineering Inc.) and Nitric Oxide (NO) assay kit (Nitrate reductase method; Nanjing Jiancheng Bioengineering Inc.) were used to measure the activity of SOD and the levels of MDA in A549 cells and the levels of NO in culture medium according to the manufacturer’s instructions, which were used to estimate antioxidants and oxidation products to explore the effect of CAE on cell redox reactions.

### Quantitative PCR

Total RNA were extracted from A549 cells and reverse transcribed to cDNA and subjected to quantitative PCR, which was performed with the BioRad CFX96 ouchTM Real-Time PCR Detection System (BioRad, CA) and threshold cycle numbers were obtained using BioRad CFX manager software. The program for amplification was 1 cycle of 95 °C for 2 min followed by 40 cycles of 95 °C for 10 s and 60 °C for 30 s. The primer sequences used in this study were 5′- TTCAGTATCACAACCTCAGCAAG-3′ (forward) and 5′- TGGACCTGCAAGTTAAAATCCC-3′ (reverse). The relative amount of iNOS gene was normalized to the amount of β-actin, and then reported as fold change of basal level.

### RNA-seq analysis

A549 cells were seeded on 10 cm culture dish and treated with 1000 µg mL^−1^ CAE in the presence of 10 ng mL^−1^ TGF-β1 (marked as CAE group), while the model group was given TGF-β1 (marked as TGF-β1 group) and the control group was added same volume of DMSO as the CAE. Cells were collected after incubation for 48 h (with 5% CO_2_ and 37 °C) and total RNA was then isolated using RNAiso Plus (Takara, Beijing, China) according to the manufacturer’s instructions. RNA-seq was performed at the Applied Protein Technology Co., Ltd in Shanghai, China. RNA-seq of each group was repeated with three independent biological replicates.

### Network pharmacology analysis

The targets of the principal components of CAE are retrieved on the Traditional Chinese Medicine Systems Pharmacology Database and Analysis Platform (TCMSP) database (https://old.tcmsp-e.com/tcmsp.php). The UniProt ID of the targets were then searched through the Universal Protein Resource (UniProt) database (https://www.uniprot.org), with the species defined as “Homo sapiens.” The targets of EMT were obtained from GeneCards (https://www.genecards.org/). Target intersection was mapped by Venny (https://bioinfogp.cnb.csic.es/tools/venny). The overlapping targets were selected and imported into the STRING database (https://string-db.org) to obtain the protein interaction relationship. The results were then imported into Cytoscape 3.9.1 software to fabricate and anatomize the Protein-Protein Interaction (PPI) Network.

### Statistical analysis

Data are expressed as the Mean ± standard error of mean (SEM). Difference between multiple groups was analyzed by one-way ANOVA with Tukey’s post-hoc tests. Difference was considered to be statistically significant when P-value < 0.05.

## Results

### CAE ameliorated LPS-induced lung injury and pulmonary fibrosis

To explore the role of CAE in the progression of LPS-induced pulmonary fibrosis, we established a pulmonary fibrosis model by intraperitoneal injection of LPS for 5 days with a dosage of 5 mg kg^−1^ day^−1^. Pre- and therapeutic treatment of CAE was indicated in Fig. 1A and B. Tissues were received after the last dosing on day 28. Body weights of mice for 28 days were recorded. CAE showed no obvious effect on body weight (Fig. 1C and D).

The lung tissue of control group showed complete alveolar structure according to H&E staining. While, the model group, which was given by LPS, had the alveolar septum thickened, many inflammatory cells infiltrated, and severe fibrous tissue proliferation observed. Compared with the model group, necrosis of lung tissue in M- and H- CAE treated groups was significantly reduced, alveolar septum was only slightly thickened, texture was close to normal, and there was only a small amount of fibrous tissue proliferation (Fig. 1E). In addition, Masson staining showed that in LPS group, collagen fiber area was greatly increased, a large number of blue collagen fibers were seen in the alveolar septum, compared with control group. Whereas the collagen fiber area in the group treated with H-CAE was significantly decreased, and very few blue collagen fibers were found in the alveolar septum (P < 0.001, Fig. 1F and H). Sirius Red staining also recorded the level of pulmonary fibrosis. It showed a larger area of Sirius Red staining in the lung tissue of LPS group than control group. Similarily, the increase in Sirius red staining induced by LPS was obviously reduced in H-CAE treatment (P < 0.001, Fig. 1G and I). However, results of Pre-treated groups (Pre L-, M- and H-CAE groups) exhibited no effects (Fig. 1E–I).

**Fig. 2:**
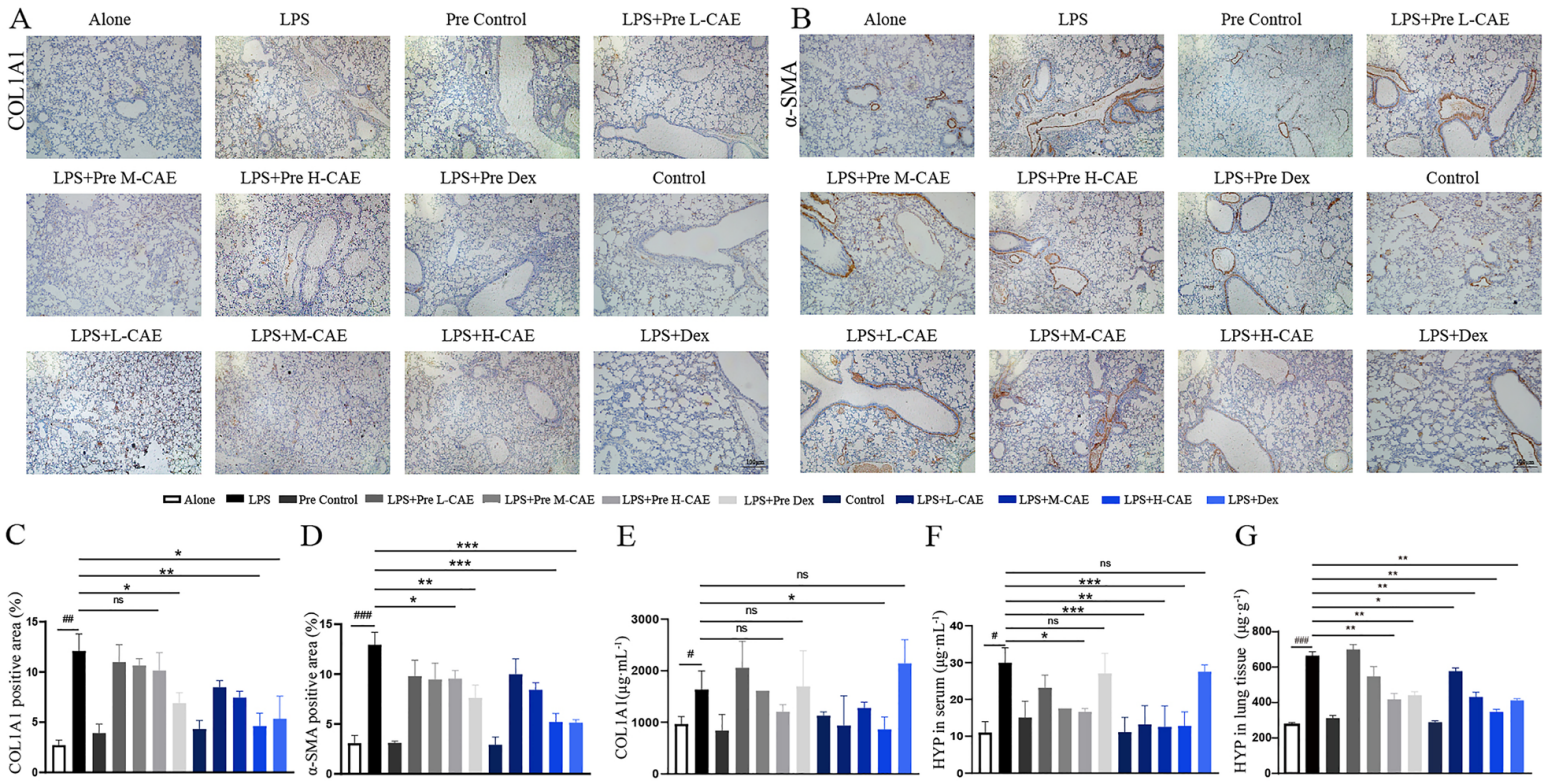
In LPS-induced pulmonary fibrosis, CAE blocked α-SMA and COL1A1 expression and amount of HYP in tissue and serum. **A**, **B** Representative immunohistochemical staining of COL1A1 and α-SMA. The scale bar is 100 μm. **C**, **D** Quantification of histological changes in different treatment groups with positive area using Image J analysis software (n = 8). **E** Quantitative analysis of COL1A1 expression (n = 8). **F**, **G** Quantitative analysis of HYP expression in serum and lung tissues (n = 8). All data were presented as means ± SEM. * P< 0.05, ** P  <0.01, ***P < 0.001 vs. the LPS group,^#^P < 0.05, ^##^P < 0.01, ^###^P < 0.001 vs. Alone group

Immunohistochemistry showed that the positive expressions of α-SMA and COL1A1 in LPS group was increased. With CAE treatment, the expression of α-SMA and COL1A1 proteins were lower and lower, especially in H-CAE group (Fig. 2A–D). To further confirm the effect of CAE on LPS-induced lung fibrosis, serum COL1A1 and hydroxyproline were analyzed. Results showed that CAE blocked the ascent of serum COL1A1 and hydroxyproline in a dose-dependent manner (Fig. 2E and F). Hydroxyproline content in lung tissues also confirmed this result (Fig. 2G). It was also worth mentioning that H-CAE was even more effective than Dex, the positive control in LPS-induced lung fibrosis. In contrast, there was no significant difference between the Pre-treated groups and LPS group (Fig. 2A–F).

### CAE improved LPS-induced pulmonary fibrosis by regulating EMT

**Fig. 3:**
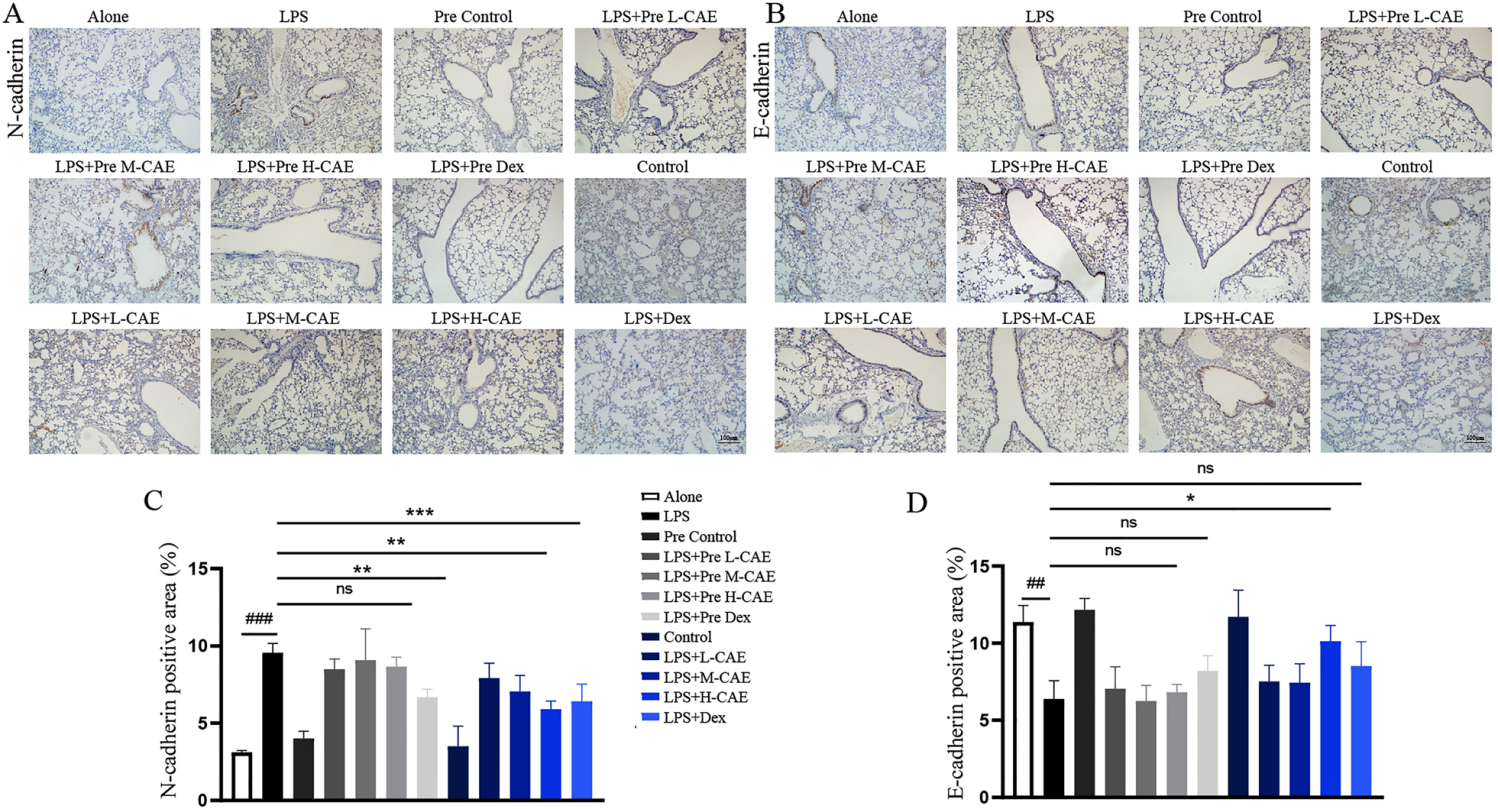
CAE reversed the expression of E-cadherin and N-cadherin in mice with pulmonary fibrosis. **A**,** B** Representative immunohistochemical staining of E-cadherin and N-cadherin. The scale bar is 100 μm. **C**, **D** Quantification of histological changes in different treatment groups with positive area using Image J analysis software (n = 8). All data were presented as means ± SEM. *P < 0.05, **P < 0.01, ***P < 1 vs. the LPS group, ^#^ P < 0.05, ^##^ P < 0.01, ^###^ P < 0.001 vs. Alone group

Immunohistochemistry staining demonstrated that E-cadherin in the lung tissues of LPS group decreased significantly, while N-cadherin increased (Fig. 3A–D). Importantly, H-CAE treatment notably reversed the expression of E-cadherin (P < 0.05) and N-cadherin (P < 0.01), which implied the reversal effect of CAE in EMT (Fig. 3A–D), whereas, pre-treated group showed no effects.

**Fig. 4:**
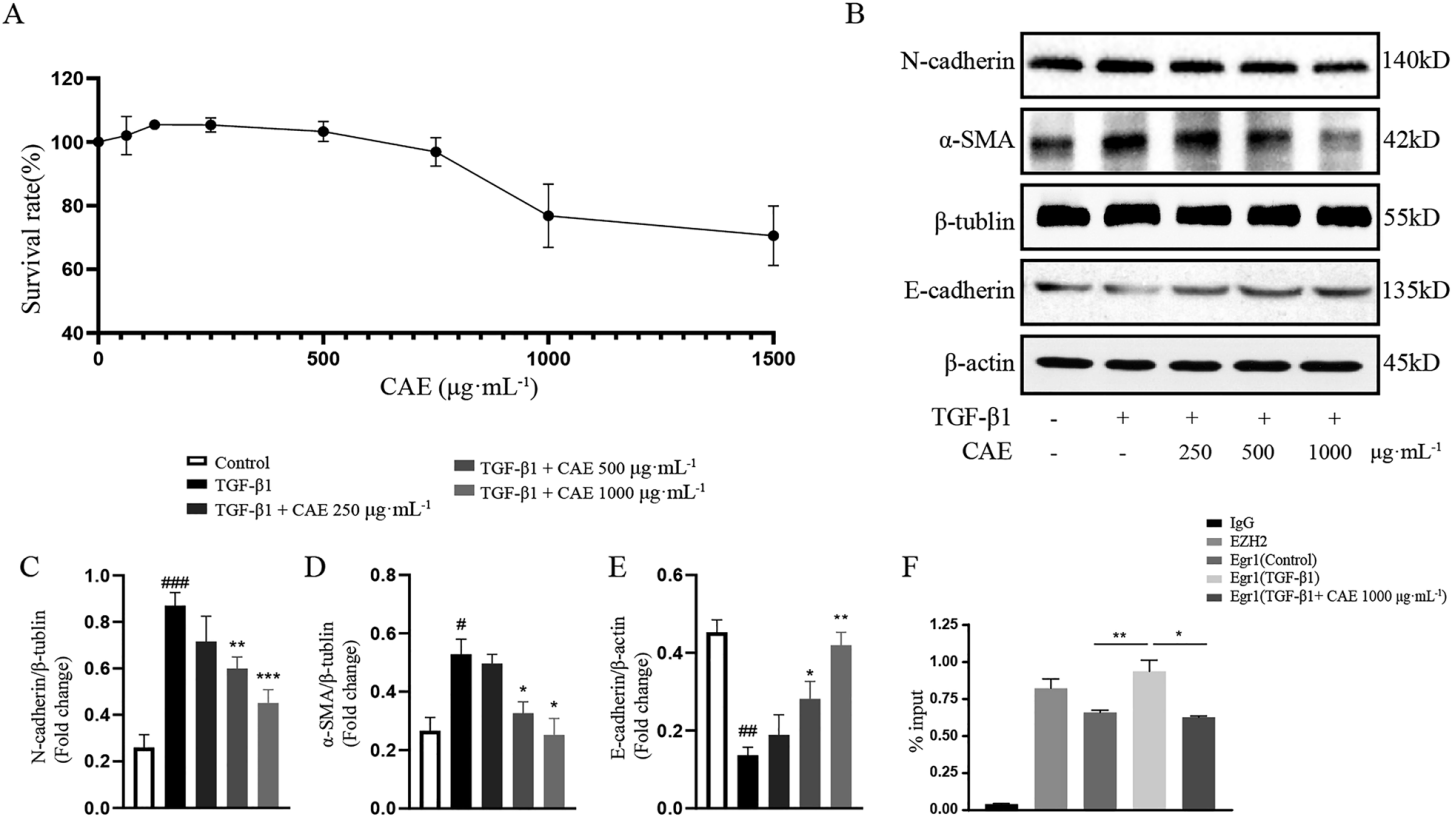
CAE inhibited TGF-β1-induced EMT in A549 cells. **A** CCK-8 experiment in A549 treated by a series dosages of CAE. **B** The protein expression of N-cadherin, α-SMA and E-cadherin were examined by western blots. **C**–**E** Quantitative analysis of N-cadherin, α-SMA and E-cadherin expression (n = 3). (F) CHIP assay of Egr1 binding COL1A1 gene promoter with EZH2 binding PTEN promoter as positive control. All data were presented as means ± SEM. *P < 0.05, **P < 0.01, ***P < 0.001 vs. the TGF-β1 group, ^#^ P < 0.05, ^##^ P < 0.01, ^###^ P < 0.001 vs. control group

**Fig. 5:**
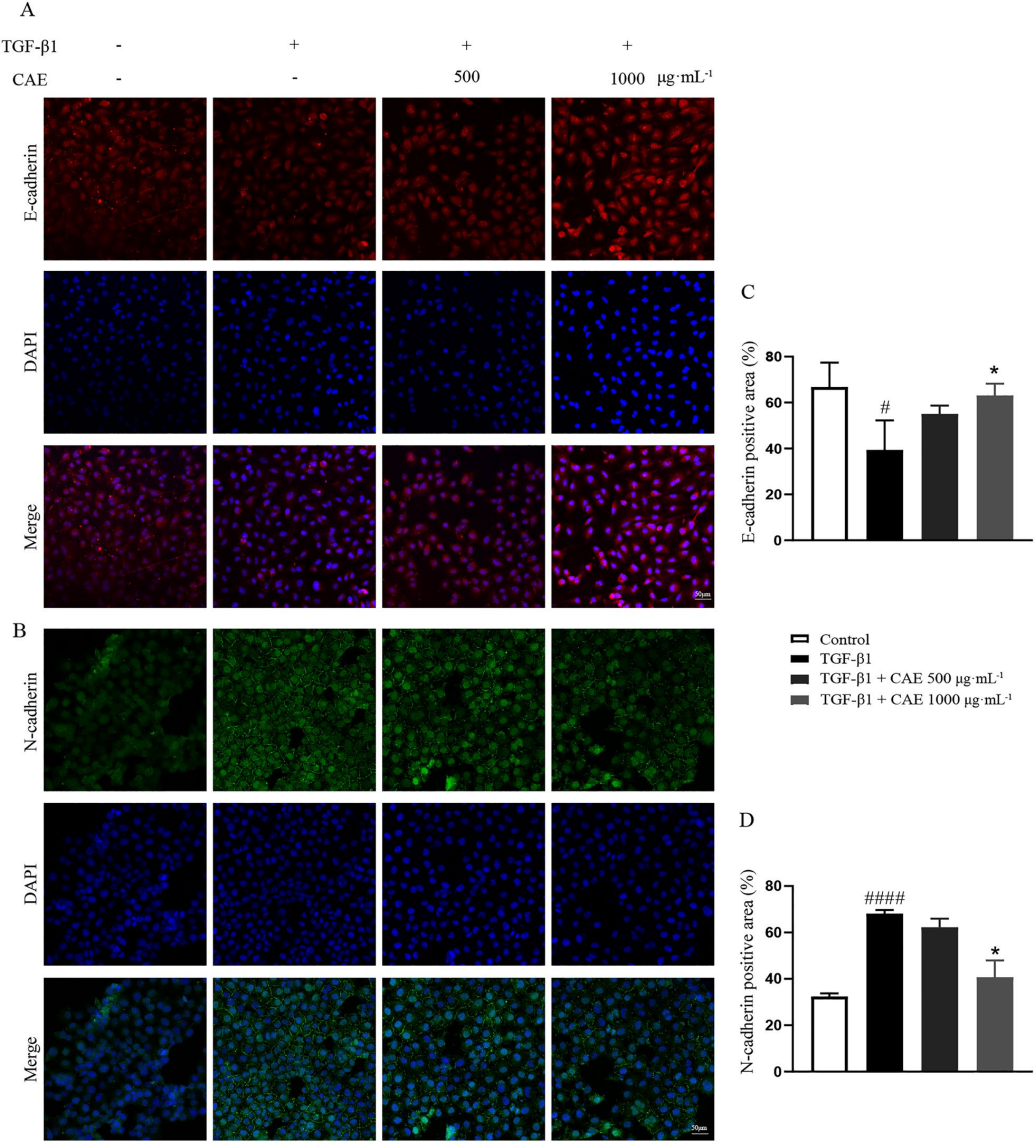
CAE increased E-cadherin and decreased N-cadherin in TGF-β1-induced A549 cells. **A** Immunofluorescence staining of A549 cells with antibodies against E-cadherin. **B** Immunofluorescence staining of A549 cells with antibodies against N-cadherin. The scale bar is 50 μm. **C**, **D** The positive staining areas were measured by Image J software (n = 3). All data were presented as means ± SEM. *P < 0.05, **P < 0.01, ***P< 0.001 vs. the TGF-β1 group, ^#^ P < 0.05, ^##^ P < 0.01, ^###^ P < 0.001 vs. control group

To clarify the effect of CAE in EMT, we determined EMT associated index in in vitro studies. CCK-8 analysis showed that CAE had no significant toxic effect in dosage of 250, 500 and 1000 µg mL^− 1^ in A549 cells (Fig. 4A) and MLE12 cells (Additional file 2A). According to the results of western blotting, CAE reversed the effects of TGF-β1 in classic EMT signals, including E-cadherin, N-cadherin and α-SMA proteins in A549 cells (Fig. 4B–E). Egr1, a protein in the AGE-RAGE pathway is an important factor in promoting EMT [19, 20]. Egr1 was found to bind to the promoter region of COL1A1 gene as a transcription factor. The binding increased under TGF-β1 stimulation and restored after CAE treatment (Fig. 4F). Immunofluorescence results also confirmed that CAE reduced the overexpression of N-cadherin and increased the depression of E-cadherin induced by TGF-β1 (Fig. 5).

### CAE inhibited EMT through the Wnt/β-catenin and STAT3/6 signaling pathway

**Fig. 6:**
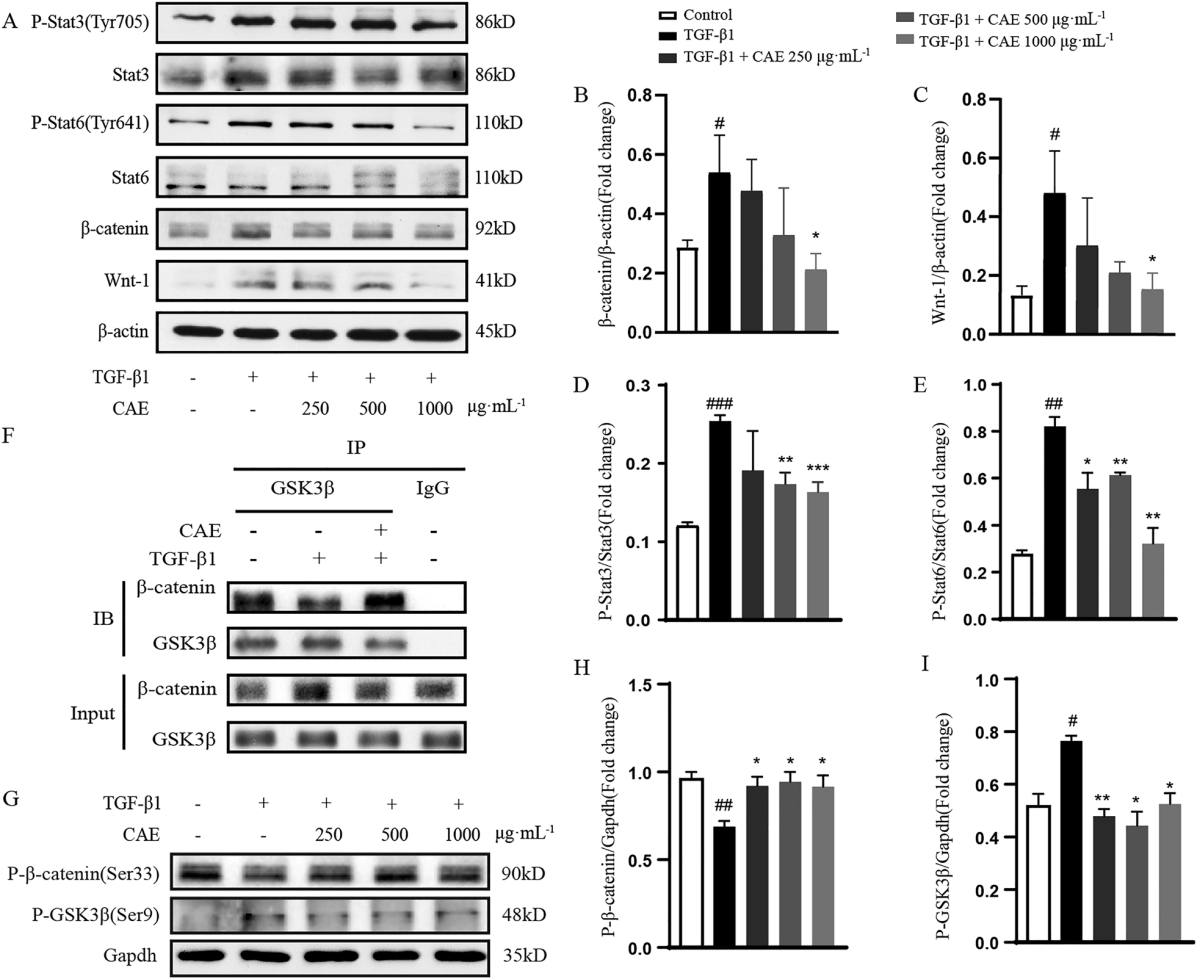
CAE reduced EMT through Wnt/β-catenin signal pathway. **A** The protein expression of β-catenin, Wnt-1, Stat3, P-Stat3, Stat6 and P-Stat6 were examined by western blots. **B**–**E** Quantitative analysis of β-catenin, Wnt-1, P-Stat3 and P-Stat6 expression (n = 3). (F) GSK3β and β-catenin immunoprecipitation experiment. **G** The protein expression of P-β-catenin (Ser33) and P-GSK3β (Ser9) were examined by western blots. (H-I) Quantitative analysis of P-β-catenin (Ser33) and P-GSK3β (Ser9) expression (n = 3). All data were presented as means ± SEM. *P < 0.05, **P < 0.01, ***P < 0.001 vs. the TGF-β1 group, ^#^ P < 0.05, ^##^ P < 0.01, ^###^ P < 0.001 vs. control group

Data further experiments revealed that CAE blocked the expression of Wnt-1 and β-catenin in A549 cells stimulated by TGF-β1, which suggested that CAE down-regulated EMT through the Wnt/β-catenin signal transduction pathway (Fig. 6A–C). The binding of β-catenin and GSK3β can form a complex and cause β-catenin to degrade. Then we found that when TGF-β1 was added, the binding of β-catenin and GSK3β reduced, while CAE treatment induced the binding of these two candidates which resulted in the degradation of β-catenin (Fig. 6F). This result was consistent with the reduction effect of CAE on β-catenin. The promotion to P-β-catenin (Ser33) and inhibition to P-GSK-3β (Ser9) of CAE also supported these results (Fig. 6G–I).

In addition, we found that P-Stat3 and P-Stat6 in the TGF-β1 stimulation group was greatly increased, and CAE exhibited inhibitory effects in Stat3/6 signals which was verified to be involved in EMT (Fig. 6A, D, E). The results of immunofluorescence showed that the levels of β-catenin, P-Stat3 and P-Stat6 in the nucleus were all increased after TGF-β1 stimulation, which could be significantly inhibited by CAE, suggested that CAE might play a critical role in the regulation of EMT (Additional file 3A–B).

### CAE partially regulated COX2/PGE2 pathway

**Fig. 7:**
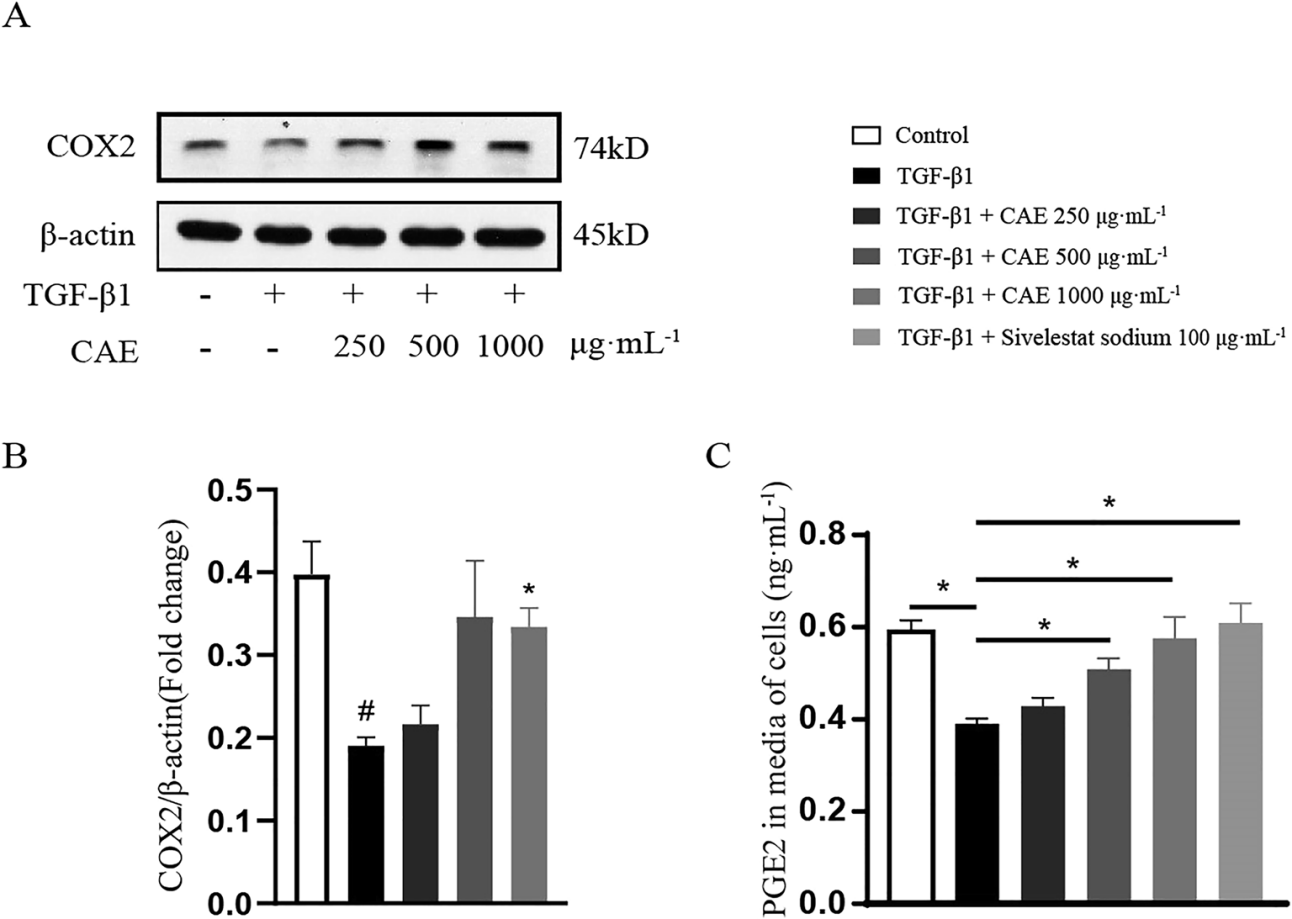
CAE partially regulated COX2/PGE2 pathway. **A** The protein expression of COX2 was examined by western blots. **B** Quantitative analysis of COX2 expression (n = 3). **C** The content of PGE2 in media of A549 cells was examined by ELISA (n = 3). All data were presented as means ± SEM. *P < 0.05, **P < 0.01, ***P < 0.001 vs. the TGF-β1 group, ^#^ P < 0.05, ^##^ P < 0.01,^###^ P < 0.001 vs. control group

We also found that TGF-β1 decreased COX2 expression, and CAE dose-dependently increased itβ (Fig. 7A, B). The result of ELISA showed that medium and high concentrations of CAE significantly restored TGF-β1-inhibited PGE2 secretion (P < 0.05, Fig. 7C). And the degree of improvement was similar to that of sivelestat sodium, which clinically used for the treatment of acute lung injury/respiratory distress syndrome with systemic inflammatory syndrome (SIRS) (Fig. 7C). These results indicated CAE had weak effect on COX2/PGE2 pathway.

### CAE reduced TGF-β1-induced oxidative stress

**Fig. 8:**
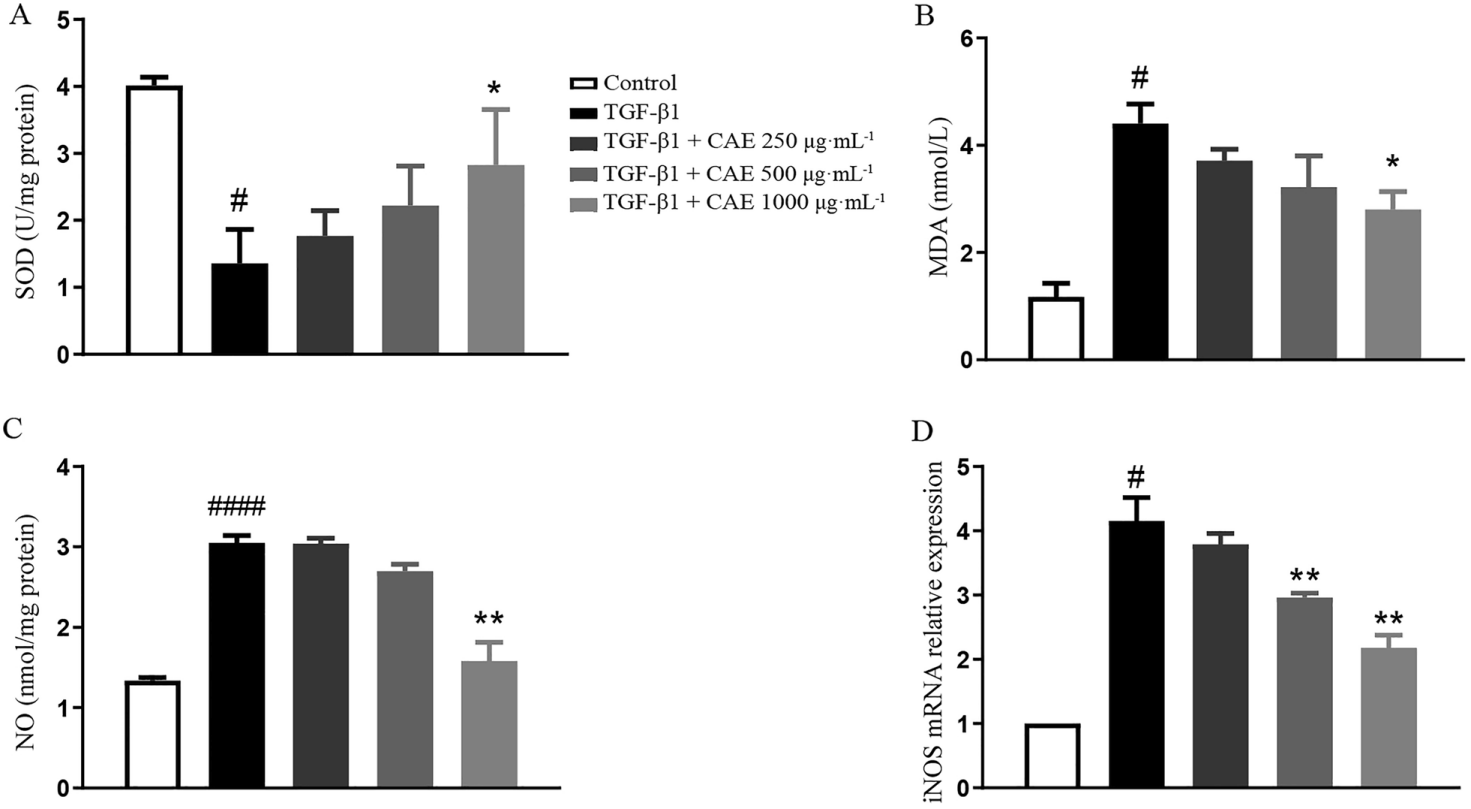
CAE decreased TGF-β1-induced oxidative stress. **A** Determination of SOD content in A549 cells. **B** Determination of MDA content in A549 cells. **C** Determination of NO production in A549 cells. **D** Determination of iNOS mRNA production in A549 cells (n = 3). All data were presented as means ± SEM. *P < 0.05, **P < 0.01, ***P < 0.001 vs the TGF-β1 group, ^#^ P < 0.05, ^##^ P < 0.01, ^###^ P < 0.001 vs control group.

To explore the effect of CAE in oxidative stress, we monitored cellular reactive oxygen species and found that CAE significantly improved the oxidative stress-induced by TGF-β1 (Fig. 8A–D), increasing SOD, decreasing MDA and iNOS mRNA, and blocking NO release.

### RNA-seq suggested the systemic impact of CAE on gene expression

**Fig. 9:**
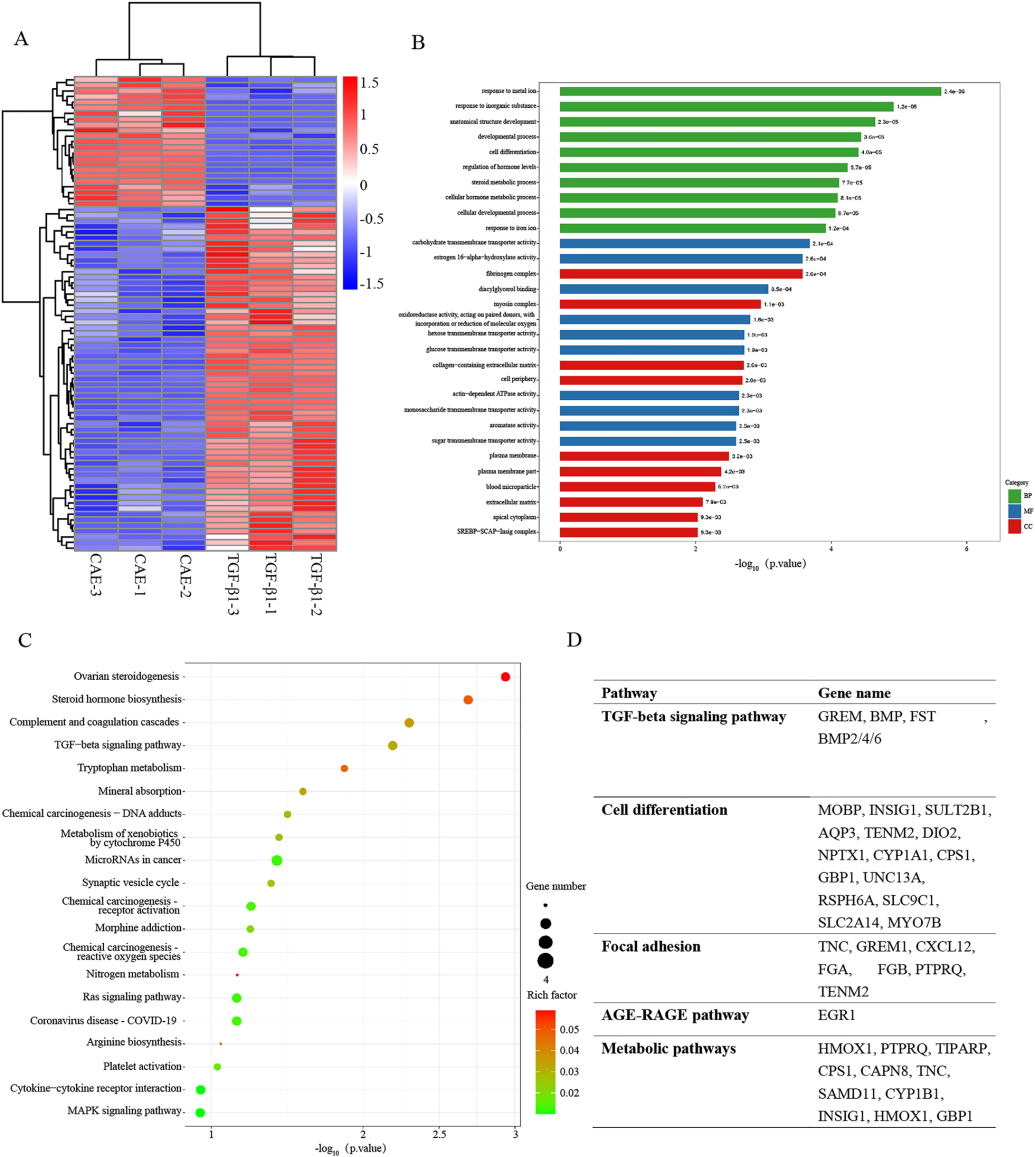
The GO classification and KEGG pathway analysis of differentially expressed genes in A549 cells treated with CAE and TGF-β1. **A** DEGs cluster analysis diagram with three replicate samples in each group. Each column represents a sample, and each row represents a gene. Red means up regulation while blue means down regulation. The above is a dendrogram of sample clustering. The closer the branches of the two samples are, the closer the expression patterns of all differential genes in the two samples are. On the left is a dendrogram of gene clusters and the closer the two gene branches are, the closer their expression levels are. **B** DEGs GO enrichment column. The abscissa is the P value after -log10 processing. The three major categories of GO are represented by different colored columns (green represents biological processes, blue represents molecular functions, and red represents cellular components). **C** Bubble chart of KEGG enrichment of DEGs. The abscissa represents the P value corresponding to the pathway. The size of the rich factor is represented by the color of the dots. The larger the value, the closer the color is to red. The number of DEGs contained in each pathway is represented by the size of the scattered dots. P value refers to the significance of pathway enrichment, the value range is [0, 1], the closer to zero, the more significant the enrichment. **D** Part of pathways and differentially expressed genes on them

To get a complete view of the gene expression affected by CAE, RNA-seq was performed using RNA extracted from TGF-β1 group or TGF-β1 + CAE group. The differentially expressed genes (DEGs) were analyzed using DESeq2. We obtained 84 DEGs, of which 23 genes were upregulated and 61 genes were downregulated in the TGF-β1 + CAE group. According to the similarity of gene expression in each sample, we performed cluster analysis of genes to visually display the expression of genes in the two groups (Fig. 9A). DEGs were subjected to Gene Ontology (GO) analysis. Based on the three aspects of biological process (BP), molecular function (MF) and cell composition (CC), we selected the most significant functional items to describe genes and gene products attributes. Through Fisher’s Exact Test, the significance of the difference between the two groups was obtained, and the functional categories of all differentially expressed proteins were found (P value < 0.05, Fig. 9B). To better understand the functions of DEGs, the involved pathways were analyzed using the Kyoto Encyclopedia of Genes and Genomes (KEGG) database. We used the KEGG pathway as the unit and the reference genome as the background, through Fisher’s Exact Test, the significance level of the gene enrichment of each pathway is analyzed and calculated, so as to determine the metabolic and signal transduction pathways that are significantly affected. The results of KEGG enrichment analysis were used scatter plots to describe (Fig. 9C). The degree of KEGG enrichment is measured by rich factor (the ratio of the number of genes located in the pathway entry among the differentially expressed genes to the total number of genes located in the pathway entry among all the annotated genes), FDR (P value corrected by BH method), and the number of genes enriched in this pathway. Compared the results of the difference between control group and the TGF-β1 group (not shown in figure) or the TGF-β1 group and the TGF-β1 + CAE group, we discovered that CAE has changed a series of gene transcription events triggered by the TGF-β signaling pathway. At the same time, the differences in gene expression in EMT-related cell differentiation, focal adhesion, AGE-RAGE pathway and metabolic pathways etc. have also aroused great interest (Fig. 9D). These changes suggested the detailed mechanism by which CAE exerts its therapeutic effect.

### Pivotal target analysis of CAE regulation of EMT

**Fig. 10:**
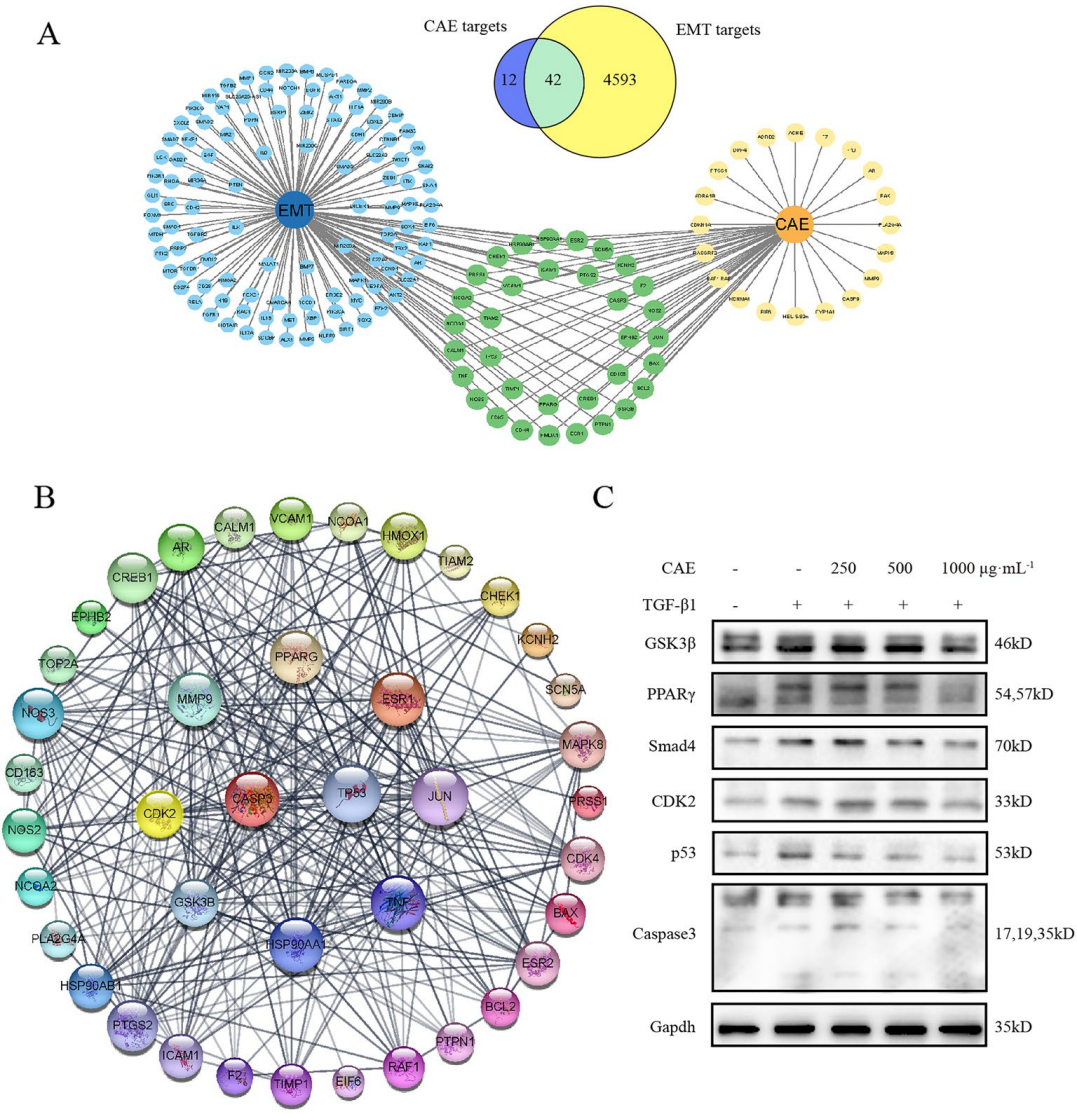
Network pharmacology analysis of CAE and EMT. **A** Main common targets of CAE and EMT. **B** The PPI of common targets. **C** CAE regulation of part of predictive vital targets

We descried 42 common targets between EMT and CAE through retrieval and analysis (Fig. 10A). We singled out the top 150 EMT targets on the website, and used Cytoscape 3.9.1 software mapping with the CAE targets to visually display the common targets of the two (Fig. 10A). PPI network of CAE in the regulation of EMT revealed that CASP3, TP53, GSK3B, JUN, PPARG and other targets were core potential targets (Fig. 10B). Western blot validated that some prime targets such as CDK2, p53, Smad4, Caspase3, PPARγ and GSK3β are regulated by CAE (Fig. 10C).

## Discussion

As we all known, ARDS, with ARDS-related pulmonary fibrosis as a common complication, is a clinical critical illness that is severely life-threatening. The occurrence of early pulmonary fibrosis indicates a higher incidence of multiple organ failure and mortality. Infection is the most common cause of ARDS, and its secondary pulmonary fibrosis has become an important cause of poor prognosis [21]. Even if patients survive, pulmonary fibrosis seriously affects the quality of life. Therefore, exploring the mechanism of ARDS-related pulmonary fibrosis and possible intervention treatments has important clinical significance for reducing the mortality of ARDS.

In this study, the method of intraperitoneal injection of LPS was used to establish a mouse ARDS-related pulmonary fibrosis model. This method has been proven to be a good model of lung injury and pulmonary fibrosis [22], which is closer to the real pathogenesis compared with bleomycin-induced model. Many Traditional Chinese Medicines have been verified to improve pulmonary injury because of multi targets and low toxicity, including Rutaceae [23, 24]. However, it is unclear about the effects of Citrus extracts in pulmonary fibrosis.

Our study suggested that therapeutic administration of CAE exhibited obvious improvement in lung inflammation and pulmonary fibrosis dose-dependently, however, preventive administration of CAE had no significant effects (Fig. 1E–I). Masson and Sirius staining showed that pre-treated groups (Pre L-, M- and H-CAE groups) exhibited no obvious effect on pulmonary fibrosis, which might indicate CAE exhibited anti-fibrotic effect by targeting activated inflammatory cells and fibroblast cells, not the resting or quiescent cells. (Fig. 1F–G). Similarly, immunohistochemical results and ELISA assay further showed only therapeutic administration of CAE ameliorated COL1A1, α-SMA expression and HYP content in fibrotic lung tissues and serum (Fig. 2). The changes of fibrotic index indicated CAE may be the potential active ingredients for Citrus in pulmonary fibrosis. It’s worth noting that in our experiments CAE pretreated groups generally did not response significantly comparing with the therapeutic CAE groups. Based on this, we speculated that CAE improved pulmonary injury and fibrosis specifically via converting activated inflammatory cells and fibroblasts from the eruptive phase to the resting phase. In addition, results showed that H-CAE had a better effect than Dex, the positive control, which was probably because of different mechanisms of CAE and Dex. Starting from traditional Chinese medicine, CAE indicates better curative effects and fewer side effects at the same time. This is of far-reaching significance in clinically eliminating the side effects of long-term Dex treatment.

EMT is a process whereby fully differentiated epithelial cells undergo transition to a mesenchymal phenotype giving rise to fibroblasts [25]. These two major cellular fates are subtly regulated and can be potently stimulated by TGF-β [26]. TGF-β1-mediated signaling in both epithelial and fibroblastic cells is a common and critical feature of fibrogenesis [27]. In this study, TGF-β1 was used to stimulate EMT in A549 cells. And our research found that CAE affected the occurrence and development of ARDS-related pulmonary fibrosis by regulating EMT, which was manifested by the expression of many EMT-related proteins (Figs. 3–5). Notably, among these related proteins, the binding of Egr1, a transcription factor, to the promoter region of the COL1A1 gene was increased under TGF-β1 stimulation and resumed under CAE treatment, which suggested a meaningful mechanism of CAE improving EMT (Fig. 4F).

E-cadherin is the core component of epithelial adherent junctions, essential for tissue development, differentiation, and maintenance. It is also fundamental for tissue barrier formation, a critical function of epithelial tissues [28]. N-cadherin expression promotes cancer metastasis, invasion, adhesion, apoptosis and angiogenesis. Elevated expression of N-cadherin is related to tumor aggressiveness [29]. The loss of epithelial feature E-cadherin and the increase of N-cadherin are important features of the occurrence and development of EMT. E-cadherin mainly exists in epithelial tissue, while N-cadherin mainly exists in muscle and fibroblasts. Interestingly, the loss of E-cadherin is a major driver or consequence of EMT, and in contrast, N-cadherin is an indicator of ongoing EMT [30, 31]. CAE achieved a dual regulation between E-cadherin and N-cadherin, which contributed to its significant effect on reverse EMT direction in pulmonary fibrosis (Figs. 3, 4B, C and E and 5).

The abnormal Wnt/β-catenin pathway activation in idiopathic pulmonary fibrosis has attracted people’s interest. Our study demonstrated that CAE significantly regulated this pathway. TGF-β1-induced Wnt-1 and β-catenin was greatly inhibited by CAE treatment. And we further verified that β-catenin degradation was due to the ascending bind of GSK3β and β-catenin by Co-Immunoprecipitation (Fig. 6). Changes in P-β-catenin and P-GSK3-β with CAE treatment also confirmed the inference (Fig. 6G–I). GSK3β regulates the transcription of E-cadherin through phosphorylation of Snail and β-catenin to trigger the proteasomal degradation. The binding of β-catenin and GSK3β can form a complex which causes β-catenin degradation [32, 33]. Therefore, Wnt/β-catenin pathway played an important role in CAE treatment. Secondly, the changes in of P-Stat3 and P-Stat6 by CAE were also distinct, CAE greatly inhibited LPS-induced P-Stat3 and P-Stat6 expression (Fig. 6A, D–E, Additional file 3), and the role of Stat3/6-related pathways was equivalently indelible.

Except of Wnt/β-catenin and STAT3/6 pathway, COX2/PGE2 signals also is involved in EMT. Current research suggested that COX2/PGE2 may regulate EMT through unconventional ways [34]. Previous studies have found that A549 developed EMT under the stimulation of TGF-β1, the extracellular matrix component increases, while COX2/PGE2 is down-regulated, and the exogenous increase of PGE2 can inhibit the EMT process with TGF-β1 treatment [35]. Our results confirmed the effect of CAE in COX2 expression and PEG2 expression (Fig. 7). Inflammation, oxidative stress and hypoxia cooperate in the induction of EMT for the progression of organ fibrosis and cancer metastasis [36]. We found that CAE caused positive changes in reactive oxygen-related indicators, which was also of great significance to the improvement of pulmonary fibrosis (Fig. 8A–D).

We predicted some conspicuous targets of CAE manipulating EMT by network pharmacology and performed experimental validation to comprehend the effect of CAE on these targets, which may be a critical mechanism for CAE to exert its therapeutic effect.

And, according to the multiple significant effect of CAE in pulmonary fibrosis and the long term clinic application of Citrus, it is meaningful to further explore the specific ingredients of CAE and potential targets of the components.

## Conclusion

In summary, CAE greatly ameliorates ARDS-related pulmonary fibrosis. And its mechanism may be related to the following factors: (1) Inhibition of EMT may be one of the mechanisms by which CAE exerts its effect on pulmonary fibrosis. (2) CAE has dual regulatory effects on E-cadherin and N-cadherin, simultaneously. (3) Based on mechanisms analysis, CAE mainly regulates Wnt/β-catenin and STAT3/6 signaling pathways to reverse EMT in pulmonary fibrosis (Fig. 10).

## Supplementary Information

**Additional file 1. ** **A**, **B** Identification of N-Methyltyramine, Synephrine, Flavanone, Hesperitin, Limonin, Narirutin, Hesperidin, Tangeretin and Sinensetin of CAE. **C** LC-MS identification of other components in CAE.

**Additional file 2.** **A** Survival rate of MLE12 cells was tested by CCK-8.

**Additional file 3. A** Immunofluorescence staining of A549 cells with antibodies against β-catenin, P-stat3and P-stat6. The scale bar is 75 μm. **B** The positive staining areas in nucleues were measured by Image J software. All data were presented as means ± SEM. *P < 0.05, **P < 0.01, ***P < 0.001 vs. the TGF-β1 group, #P < 0.05, ##P < 0.01, ###P < 0.001 vs. control group.

## Data Availability

The datasets generated and/or analysed during the current study are available in the Mendeley Data repository, DOI: 10.17632/7xk27ntvsn.2.

## Abbreviations

ARDS: Acute respiratory distress syndrome
LPS: Lipopolysaccharide
CAE: Citrus alkaline extracts
EMT: Epithelial-mesenchymal transition
STAT: Signal transducer and activator of transcription
COX2: Cyclooxygenase 2
PEG2: Prostaglandin E2
TGF-β1: Transforming growth factor β1
Dex: Dexamethasone
CCK-8: Cell counting Kit-8
DMEM: Dulbecco’s modified eagle medium
SOD: Superoxide dismutase
MDA: Malondialdehyde
NO: Nitric oxide
PVDF: Poiyvinylidene fluoride
IHC: Immunohistochemistry staining
α-SMA: α-smooth muscle actin
FBS: Fetal bovine serum
PBS: Phosphate buffered saline
COL1A1: Collagen type I alpha 1 chain
GSK3β: Glycogen synthase kinase 3β
HRP: Horseradish peroxidase
Egr1: Early growth response 1
SDS-PAGE: Sodium dodecyl sulfate-polyacrylamide gel electrophoresis
DMSO: Dimethyl sulfoxide
HYP: Hydroxyproline
TCMSP: Traditional chinese medicine systems pharmacology database and analysis platform
KEGG: Kyoto encyclopedia of genes and genomes
DEGs: Differentially expressed genes
GO: Gene ontology
BP: Biological process
MF: Molecular function
CC: Cell composition
CDK2: Cyclin dependent kinase 2
Smad4: Smad family member 4
PPARγ: Peroxisome proliferator-activated receptor γ
