# Supplementary material for: Citrus alkaline extracts improve LPS-induced pulmonary fibrosis via epithelial mesenchymal transition signals
Source: Chin Med. 2023 May 29;18:62. doi: 10.1186/s13020-023-00766-0 (PMC10226443; doi:10.1186/s13020-023-00766-0)
Supplement: Supplementary file 1 — Additional file 1. A, B Identification of N-Methyltyramine, Synephrine, Flavanone, Hesperitin, Limonin, Narirutin, Hesperidin, Tangeretin and Sinensetin of CAE. C LC-MS identification of other components in CAE. [file 13020_2023_766_MOESM1_ESM.docx]

**Additional file 1.** Identification of N-Methyltyramine, Synephrine, Flavanone, Hesperitin, Limonin, Narirutin, Hesperidin, Tangeretin and Sinensetin of CAE.

1. Standards.
2. Standards of Synephrine, N-Methyltyramine, Flavanone, Sinensetin, Tangeretin, Hesperidin and Limonin.

1. Standards of Narirutin, Naringin, Hesperidin and Neohesperidin.

1. Synephrine
2. N-Methyltyramine
3. Flavanone

1. Sinensetin (8.672) and Tangeretin (9.589)

1. Hesperidin

1. Limonin

1. Narirutin (3.353) and Naringin (3.900)

1. Hesperidin (4.245) and Neohesperidin (4.767)

1. Compounds in CAE

1. Synephrine in CAE

1. N-Methyltyramine in CAE

1. Flavanone in CAE

1. Isomer of Sinensetin (8.282), Sinensetin (8.668) and Tangeretin (9.579) in CAE

1. Hesperidin (9.066) in CAE

1. Limonin in CAE

1. Narirutin in CAE

1. Hesperidin in CAE

1. LC-MS identification of other components in CAE.

| Component | Formula |
| --- | --- |
| LINDERANE  Complanatoside  Narcissoside  Isorhamnetin-3-O-neohespeidoside  Hesperetin  Adenosine  Anisaldehyde  4'-Hydroxyacetophenone  Oxidized procurin  BRAZILIN  Butylparaben  Isoliquiritigenin  Dihydrodaidzein  Liquiritigenin  Pinocembrin  1,7-dimethoxyxanthone  Coumarin  Eriodictyol  PolygalaxanthoneⅣ  neoeriocitrin  4-Dimethylaminobenzaldehyde  Nobiletin  Engeletin  Astilbin  Methylophiopogonanone A  BRAZILIN  Eriodictyol  Germacrone;  (3E,7E)-3,7-dimethyl-10-propan-2-ylidenecyclodeca-3,7-dien-1-one  Aristolone  Alpha-Cyperone  Pulegone  Camphor;  Astilbin  Ferulic Acid  Isoferulic acid  Isoliquiritigenin  pinocembrin  Dihydrodaidzein  Liquiritigenin  1,7-dimethoxyxanthone  PolygalaxanthoneⅣ  Neoeriocitrin  Ethyl 4-hydroxybenzoate  Paeonol  Naringenin  Engeletin  Complanatoside  Narcissoside  Isorhamnetin-3-O-neohespeidoside  7-Methoxycoumarin  Hymecromone  7-Hydroxycoumarin  4-Hydroxycoumarin  4-Dimethylaminobenzaldehyde  Nomilin | C_15_H_16_O_4_  C_28_H_32_O_16_  C_28_H_32_O_16_  C_28_H_32_O_16_  C_16_H_14_O_6_  C_10_H_13_N_5_O_4_  C_8_H_8_O_2_  C_8_H_8_O_2_  C_16_H_14_O_5_  C_16_H_14_O_5_  C_11_H_14_O_3_  C_15_H_12_O_4_  C_15_H_12_O_4_  C_15_H_12_O_4_  C_15_H_12_O_4_  C_15_H_12_O_4_  C_9_H_6_O_2_  C_15_H_12_O_6_  C_27_H_32_O_15_  C_27_H_32_O_15_  C_9_H_11_NO  C_21_H_22_O_8_  C_21_H_22_O_10_  C_21_H_22_O_11_  C_19_H_18_O_6_  C_16_H_14_O_5_  C_15_H_12_O_6_  C_15_H_22_O  C_15_H_22_O  C_15_H_22_O  C_10_H_16_O  C_10_H_16_O  C_21_H_22_O_11_  C_10_H_10_O_4_  C_10_H_10_O_4_  C_15_H_12_O_4_  C_15_H_12_O_4_  C_15_H_12_O_4_  C_15_H_12_O_4_  C_15_H_12_O_4_  C_27_H_32_O_15_  C_27_H_32_O_15_  C_9_H_10_O_3_  C_9_H_10_O_3_  C_15_H_12_O_5_  C_21_H_22_O_10_  C_28_H_32_O_16_  C_28_H_32_O_16_  C_28_H_32_O_16_  C_10_H_8_O_3_  C_10_H_8_O_3_  C_9_H_6_O_3_  C_9_H_6_O_3_  C_9_H_11_NO  C_28_H_34_O_9_ |
