# Supplementary figures and images for: Citrus alkaline extracts improve LPS-induced pulmonary fibrosis via epithelial mesenchymal transition signals
Source: Chin Med. 2023 May 29;18:62. doi: 10.1186/s13020-023-00766-0 (PMC10226443; doi:10.1186/s13020-023-00766-0)

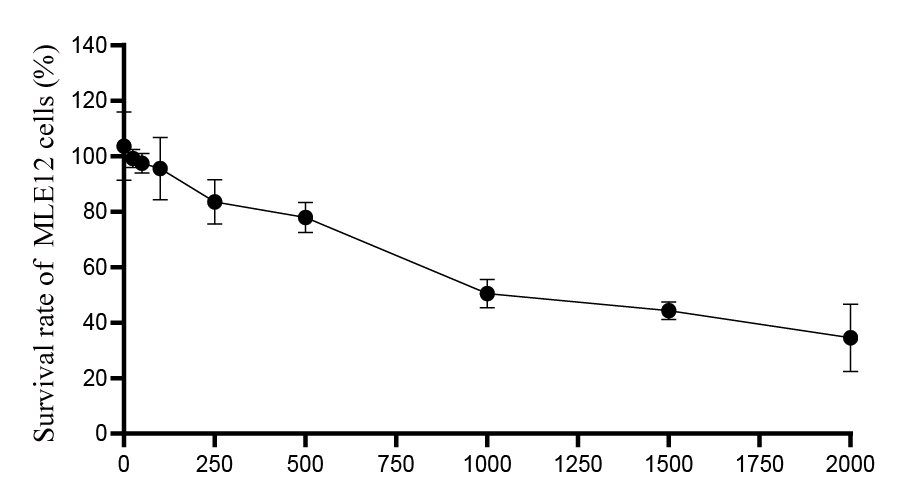

Supplement: Supplementary file 2 — Additional file 2. A Survival rate of MLE12 cells was tested by CCK-8. [file 13020_2023_766_MOESM2_ESM.jpg]

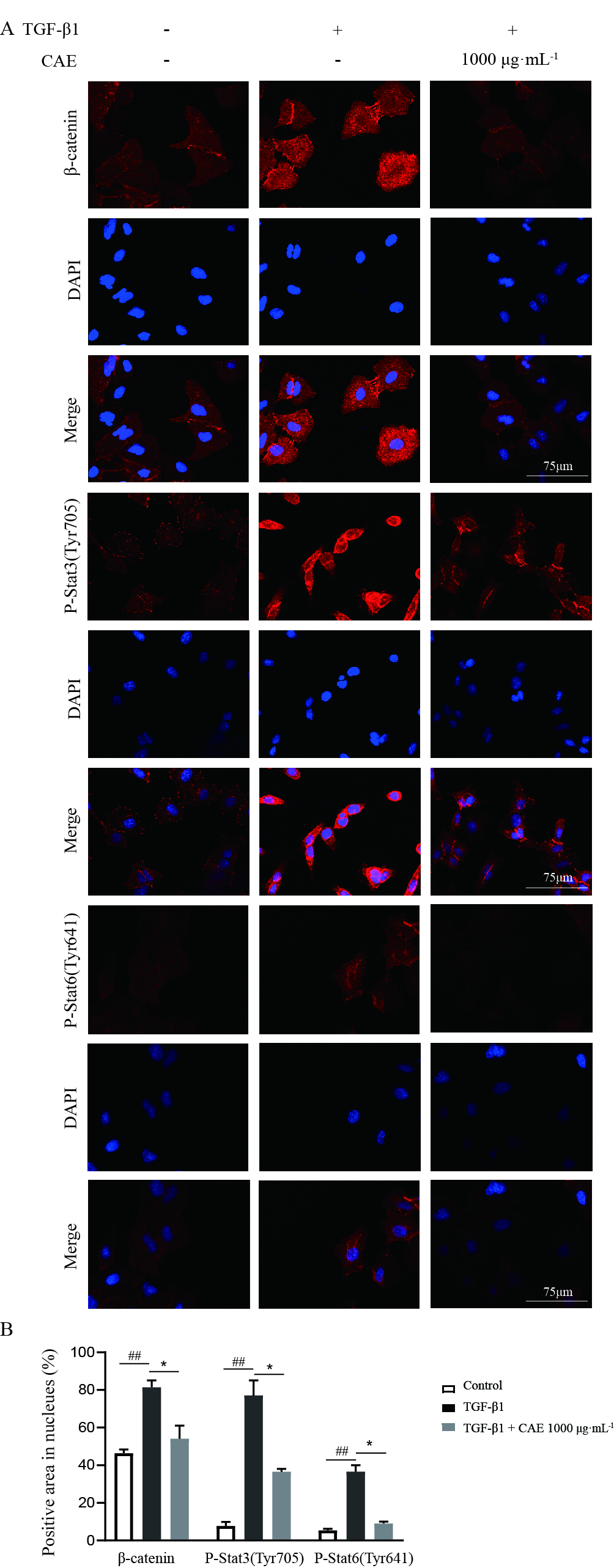

Supplement: Supplementary file 3 — Additional file 3. A Immunofluorescence staining of A549 cells with antibodies against β-catenin, P-stat3and P-stat6. The scale bar is 75 μm. B The positive staining areas in nucleues were measured by Image J software. All data were presented as means ± SEM. *P < 0.05, **P < 0.01, ***P < 0.001 vs. the TGF-β1 group, #P < 0.05, ##P < 0.01, ###P < 0.001 vs. control group. [file 13020_2023_766_MOESM3_ESM.jpg]
